# Brassinosteroid signaling in plant development and adaptation to stress

**DOI:** 10.1242/dev.151894

**Published:** 2019-03-14

**Authors:** Ainoa Planas-Riverola, Aditi Gupta, Isabel Betegón-Putze, Nadja Bosch, Marta Ibañes, Ana I. Caño-Delgado

**Affiliations:** 1 Department of Molecular Genetics, Centre for Research in Agricultural Genomics (CRAG) CSIC-IRTA-UAB-UB, Barcelona E-08193, Spain; 2 Departament de Física de la Matèria Condensada, Universitat de Barcelona, Barcelona 08028, Spain; 3 Universitat de Barcelona Institute of Complex Systems (UBICS), Universitat de Barcelona, Barcelona 08028, Spain

**Keywords:** Brassinosteroid, Growth, Root, Stem cell, Stress

## Abstract

Brassinosteroids (BRs) are steroid hormones that are essential for plant growth and development. These hormones control the division, elongation and differentiation of various cell types throughout the entire plant life cycle. Our current understanding of the BR signaling pathway has mostly been obtained from studies using *Arabidopsis thaliana* as a model. In this context, the membrane steroid receptor BRI1 (BRASSINOSTEROID INSENSITIVE 1) binds directly to the BR ligand, triggering a signal cascade in the cytoplasm that leads to the transcription of BR-responsive genes that drive cellular growth. However, recent studies of the primary root have revealed distinct BR signaling pathways in different cell types and have highlighted cell-specific roles for BR signaling in controlling adaptation to stress. In this Review, we summarize our current knowledge of the spatiotemporal control of BR action in plant growth and development, focusing on BR functions in primary root development and growth, in stem cell self-renewal and death, and in plant adaption to environmental stress.

## Abstract (precis)

**Summary:** This Review summarizes current knowledge of the spatiotemporal control of brassinosteroid function in plants, focusing on primary root development and growth, stem cell self-renewal and death, and adaptation to environmental stress.

## Introduction

Brassinosteroids (BRs) are phytohormones that were originally discovered in *Brassica napus* pollen based on their ability to promote growth (Mitchell et al., 1970). Since their discovery, the main components of the canonical BR signaling pathway have been identified through multiple genetic and biochemical screens (Vert et al., 2005; Zhu et al., 2013). BR perception occurs at membrane-localized receptors and downstream cytosolic regulators transduce BR-mediated signals to the nucleus where they activate the transcription of BR-responsive genes that drive cellular growth (Belkhadir and Jaillais, 2015; Zhao and Li, 2012). Accordingly, mutations in genes encoding the main components of the BR synthesis and signaling pathways result in severe dwarfism, impaired organ growth and development, and limited plant fertility and yield (Li and Chory, 1997; Singh and Savaldi-Goldstein, 2015). Despite such knowledge of BR pathway components, many questions remain unclear, including how BRs function in a cell-specific manner, how the BR pathway interacts with other hormonal pathways under normal and environmentally challenging scenarios, and in which tissues BR synthesis occurs (Caño-Delgado and Blázquez, 2013; Vukasinovic and Russinova, 2018).

Over the past few decades, BR hormones have been shown to be essential for cell elongation and, as such, initial studies on hypocotyl elongation have been very rewarding in terms of understanding the transcriptional responses that trigger elongation (Clouse and Sasse, 1998). However, since the discovery that BRs also play a role in cell division (González-García et al., 2011; Hacham et al., 2011), studies have switched focus in an attempt to understand how BRs modulate growth and development in plants, using the primary root of *Arabidopsis thaliana* as a model. In this context, techniques such as fluorescence-activated cell sorting (Brady et al., 2007), and tools that allow the local expression of signaling components (Marquès-Bueno et al., 2016) and the visualization of cell-specific protein-protein interactions (Long et al., 2017), have been instrumental in elucidating novel BR signaling components and cell-specific signals (Fàbregas et al., 2013; Vilarrasa-Blasi et al., 2014; Vragović et al., 2015). More recent work on BRs has also begun to decode the mechanisms by which BR-mediated signaling regulates adaptation to biotic (De Bruyne et al., 2014) and abiotic (Lozano-Durán and Zipfel, 2015; Nolan et al., 2017a) stresses. Here, we review these recent advances that aim to decipher the spatiotemporal control of BR action. First, we provide an overview of the BR signal transduction pathway and then discuss how BRs regulate root growth and development in a cell-specific fashion. We also highlight how BRs function within some of the most special cells of the plant, the root stem cells. Finally, we review our current understanding of the roles of BRs and their crosstalk with other hormones in mediating adaptation to abiotic stresses, such as drought, temperature changes and salinity.

## Brassinosteroid ligand perception and signal transduction

**Fig. 1.**
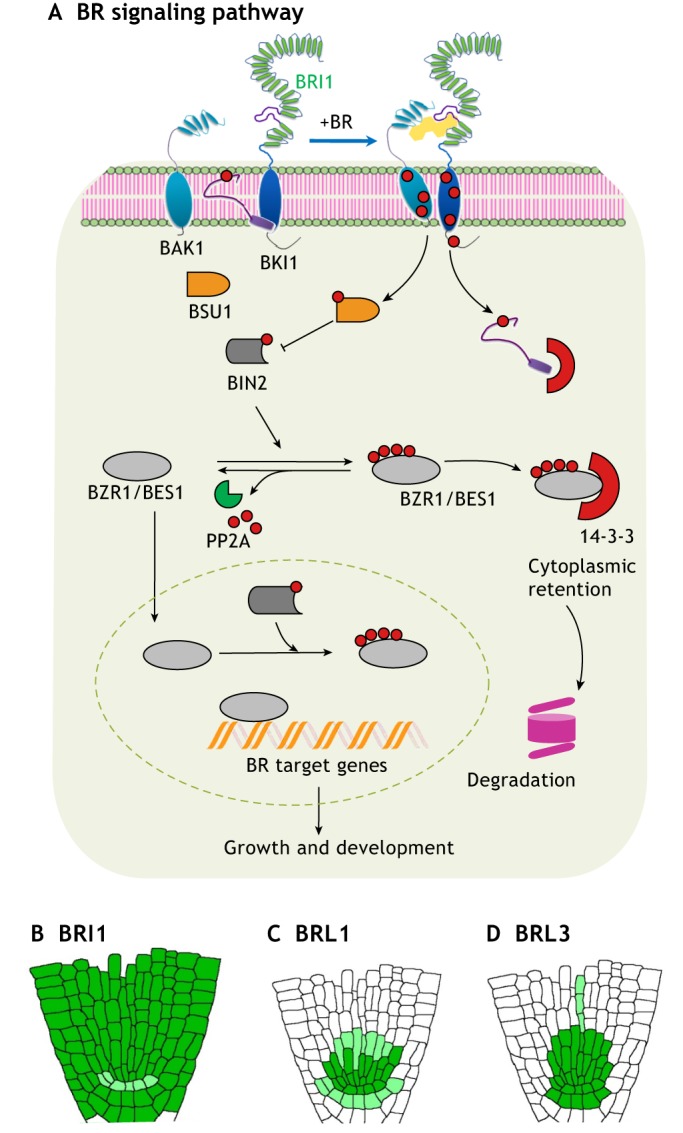
**An overview of the BR signaling pathway.** (A) Schematic of the BRI1 signaling pathway. In the absence of BR, BIN2 phosphorylates BZR1 and BES1 proteins, inactivating them by promoting their binding to 14-3-3 proteins, leading to their cytoplasmic retention and degradation. When BRI1 perceives BR molecules, it heterodimerizes with BAK1, initiating an intracellular phosphorylation relay cascade that ends with the dephosphorylation and consequent activation of BZR1 and BES1. (B-D) Schematics of the root tissue-specific expression of BR receptors. BRI1 is expressed throughout the root (B), whereas BRL1 (C) and BRL3 (D) exhibit a more discrete expression pattern, being active mainly in the root stem cell niche area. Dark green represents high expression of the protein, whereas light green represents lower expression.

BR hormones are perceived extracellularly by members of the BRI1 (BRASSINOSTEROID INSENSITIVE 1) leucine-rich repeat receptor-like kinase (LRR-RLK) family (Li and Chory, 1997; Wang et al., 2001). The BR hormone binds directly to a 93-amino-acid region located within the extracellular domain of membrane-bound BRI1 (Hothorn et al., 2011; Kinoshita et al., 2005; Sun et al., 2013). Direct binding triggers the formation of a BRI1-BAK1 [BRASSINOSTEROID INSENSITIVE 1-ASSOCIATED RECEPTOR KINASE 1, also known as SERK3 (SOMATIC EMBRYOGENESIS RECEPTOR KINASE 3)] heterodimer, which in turn initiates an intracellular phosphorylation relay cascade (Li and Nam, 2002; Russinova et al., 2004). The cascade (Fig. 1A) culminates in promotion of the activity and stability of the plant-specific transcription factors BZR1 (BRASSINAZOLE RESISTANT 1) (Wang et al., 2002) and BES1 (BRI1-EMS-SUPPRESSOR 1) (Yin et al., 2002), which directly control the transcription of thousands of BR-responsive genes and hence regulate a plethora of developmental events in the plant (He et al., 2002; Sun et al., 2010). When BRs are absent, the GSK3-like kinase BIN2 (BRASSINOSTEROID-INSENSITIVE 2) phosphorylates BZR1/BES1 proteins and inactivates them, promoting their binding to 14-3-3 proteins and leading to their cytoplasmic retention and degradation (Gampala et al., 2007; Li and Nam, 2002; Peng et al., 2008). This thereby inhibits their ability to bind DNA and causes pathway inactivation.

Based on the presence of the extracellular BR-binding domain, there are three membrane-localized BRI1-like homologs named BRL1, BRL2 and BRL3 (BRI1-LIKE 1, 2 and 3). Whereas BRL1 and BRL3 are functional BR receptors that, like BRI1, can bind to steroid molecules with high affinity, BRL2 appears not to be a functional BR receptor (Caño-Delgado et al., 2004). Furthermore, whereas BRI1 is expressed nearly ubiquitously in the root (Friedrichsen and Chory, 2001) (Fig. 1B), the BRLs are found only in some specific tissues (Fig. 1C,D). For example, BRL1 and BRL3 are localized in vascular stem cells, where they govern cell-specific BR-response pathways (Caño-Delgado et al., 2004; Fàbregas et al., 2013; Salazar-Henao et al., 2016). Under native conditions, both BRL1 and BRL3 can heterodimerize with the BAK1 co-receptor, but not with BRI1, and form a complex (Fàbregas et al., 2013). These studies suggest that BRI1 and the BRLs are able to form different receptor complexes in different cell types, thereby performing different signaling roles, but the specific downstream components of the BRL1 and BRL3 pathways remain unknown.

## The primary root as a model for deciphering cell-specific brassinosteroid signaling

**Fig. 2.**
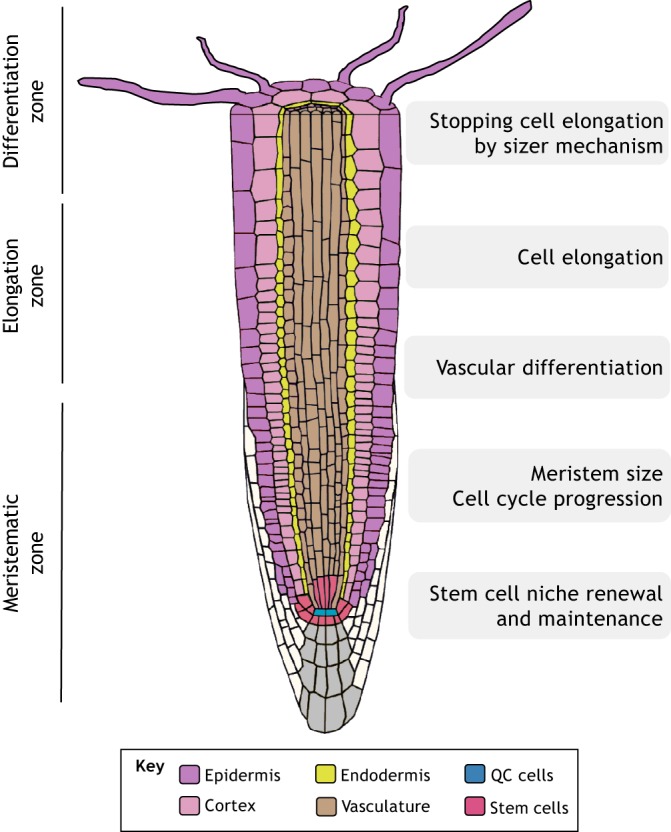
**BR functions in the primary root.** BRs are involved in a variety of cell-specific processes that occur within the different zones of the root. These include processes such as cell cycle division, cell elongation and cell differentiation.

Owing to its simple and radial organization of cell types, the primary root of *Arabidopsis* provides an excellent model for dissecting signaling mechanisms with cell-specific resolution (Dolan et al., 1993; Scheres et al., 1994). Indeed, a number of studies of the primary root have shown that BRs control specific cellular processes in distinct root cell types (Fig. 2).

BRs play an important role in overall root development; both an excess and a lack of BRs are detrimental to primary root growth and development. On the one hand, mutants lacking BR compounds or BR receptors exhibit short roots, indicating that BRI1 signaling is required for root growth (Chaiwanon and Wang, 2015; González-García et al., 2011; Hacham et al., 2011; Mussig et al., 2003). On the other hand, short roots are also observed in *bes1-D* (gain-of-function) mutants, or in plants treated with high concentrations of BRs (González-García et al., 2011; Mussig et al., 2002). The short roots of mutants with impaired BR biosynthesis can be rescued by treatment with low concentrations of BR (Chaiwanon and Wang, 2015). Moreover, supporting the notion that BRs can promote root growth, it has been shown that wild-type roots treated with low concentrations of BRs increase their length (González-García et al., 2011; Mussig et al., 2003), although this enlargement is small and not always detectable (Chaiwanon and Wang, 2015). Altogether, these results suggest that, rather than controlling root growth in a linear fashion, the correct balance of BR levels appears to be crucial for normal root growth and development (González-García et al., 2011).

Root growth also depends on cell proliferation at the meristem and on cell elongation prior to differentiation. BRs impinge on both of these processes. BRs modulate meristematic proliferation (González-García et al., 2011; Hacham et al., 2011) and have been proposed as key regulators in the optimal control of cell cycle progression (González-García et al., 2011). BRs have been also proposed to be crucial for optimal cell expansion (Chaiwanon and Wang, 2015; Clouse and Sasse, 1998). Recent mathematical and computational modeling has further demonstrated that root growth features depend on the mechanism by which cell elongation terminates, e.g. whether cells stop elongating according to their spatial position along the root, according to a time interval, and/or according to their cell size (Pavelescu et al., 2018). Quantification of cell length in single roots, together with mathematical and computational modeling, suggests that the dominant mechanism for cell elongation termination is a size-based mechanism whereby root cells stop expanding when they reach a determined length, and that BRI1 facilitates this mechanism (Pavelescu et al., 2018). In addition, this suggests that BR signaling at least partially controls these three separate functions: cell division, cell elongation rate and termination of cell elongation (Pavelescu et al., 2018). Indeed, plants treated with high concentrations of BR increase expansion at the meristem and reduce the number of meristematic cells, but do not exhibit an increase in meristem cell length (Chaiwanon and Wang, 2015).

The control of root growth by BR signaling is also spatially segregated throughout the root. BR signaling is not found homogeneously throughout the root, with BZR1 being more strongly activated at the transition from the meristem to the elongation zones and in the elongation zone itself (Chaiwanon and Wang, 2015). Moreover, BR signaling induces target genes in the epidermis (the outer layer of the root) but mostly represses genes in the stele (the inner layer) (Vragović et al., 2015), highlighting that BR signaling can elicit tissue-specific responses. Based on these results, it has been proposed that BR signaling can function in a non-cell-autonomous manner, signaling from the epidermis to inner cells (Hacham et al., 2011; Vragović et al., 2015). Interestingly, the differential expression of BRI1 between hair and non-hair epidermal cells controls the length of mature cells as well as their sensitivity to BR hormonal treatment (Fridman et al., 2014). Furthermore, it was recently shown that expressing BRI1 under the control of cell-specific promoters of the protophloem (a component of the stele) such as pMAKR5 (MEMBRANE-ASSOCIATED KINASE REGULATOR 5) and pCVP2 (COTYLEDON VASCULAR PATTERN 2) rescues the phenotypic defects of *bri1 brl1 brl3* triple receptor mutants, suggesting that a phloem-derived signal can non-autonomously drive root growth (Kang et al., 2017). These results point to the complexity of BR signaling and highlight some level of directionality – from inner to outside cell layers and vice versa – of BR signaling in the root. This signaling directionality likely depends on the cell-specific expression and site of action of BR receptors, which could promote specific signals and thus contribute differentially to overall root development. Given that BRL receptors function in the phloem (Caño-Delgado et al., 2004) and the recent proposed role for BRL3 in root mobilization of osmoprotectant metabolites to confer drought resistance (Fàbregas et al., 2018), we propose that that BR receptors expressed in the inner layers of the root may selectively promote growth under stress.

BR signaling is also involved in the development of vascular tissues within the plant. Early studies in *Zinnia elegans* cells indicate that BR synthesis increases prior to, and is necessary for, tracheary element differentiation (Yamamoto et al., 2001), and in *Arabidopsis* suspension cultures BRs induce VND7-mediated xylem cell wall differentiation (Yamaguchi et al., 2010). In *Arabidopsis*, BR-deficient plants harboring mutations in genes such as *CPD* (*CONSTITUTIVE PHOTOMORPHOGENIC DWARF*) and *DWF7* (*DWARF 7*) have abnormal xylem development (Choe et al., 1999; Szekeres et al., 1996). BR receptor mutants also exhibit abnormal vascular differentiation, a process in which BRI1 and the BRLs have redundant functions (Caño-Delgado et al., 2004). In the primary root, BR suppresses radial vascular cell divisions (Fàbregas et al., 2013; Kang et al., 2017). In line with this, the *brl1 brl3 bak1-3* triple mutant is hypersensitive to BR in the stele, showing greater stele narrowing than that of wild-type, *bak1* or *brl1 brl3* mutant plants upon BR treatment (Fàbregas et al., 2013). In addition, the wider stele of the *bri1 brl1 brl3* triple mutant increases when BRI1 is expressed in the stele and decreases when BRI1 is expressed in the epidermis (Kang et al., 2017). Thus, the control of formative asymmetric divisions in the stele can be controlled both cell-autonomously and non-cell-autonomously in an opposite manner, implying that the nature of the stele divisions might depend on the localization of the instructing signal. Conversely, the control of formative asymmetric cell divisions in the epidermis appears to be cell-autonomous, as expression of BRI1 in the epidermis restores the wider phenotype of the *bri1 brl1 brl3* triple receptor mutant (Kang et al., 2017). Of note, BRs together with auxins are also involved in establishing the periodic pattern of vascular bundles in the *Arabidopsis* shoot (Ibanes et al., 2009); the quantification of this pattern, together with mathematical modeling, supports the notion that cell numbers, which are controlled by BRs, are relevant for vascular patterning. However, despite these various lines of evidence linking BRs and vascular development, little is known about the contribution of different BR receptors and downstream transcriptional players in the formation of functional vascular tissues and overall organ growth.

## The role of brassinosteroid signaling in stem cell self-renewal and differentiation

The root stem cell niche comprises a small group of stem cells located at the base of the meristem in the root apex. These cells are essential for sustaining root growth, as they continuously provide the precursors of more-specialized cells, and to replace tissues that have been damaged (Dolan et al., 1993; Sabatini et al., 2003; van den Berg et al., 1997). The core of the niche contains a group of cells with very low mitotic activity that are collectively known as the quiescent center (QC). The QC maintains the undifferentiated state of the surrounding stem cells (Sarkar et al., 2007; van den Berg et al., 1997) whilst maintaining its own stemness, but it can also act as a reservoir of cells that can replenish damaged ones (Heyman et al., 2013; Vilarrasa-Blasi et al., 2014). As we discuss below, BRs play a key role in maintaining the identity and quiescence of QC cells (González-García et al., 2011), and thereby affect the maintenance of the root stem cell niche.

**Fig. 3.**
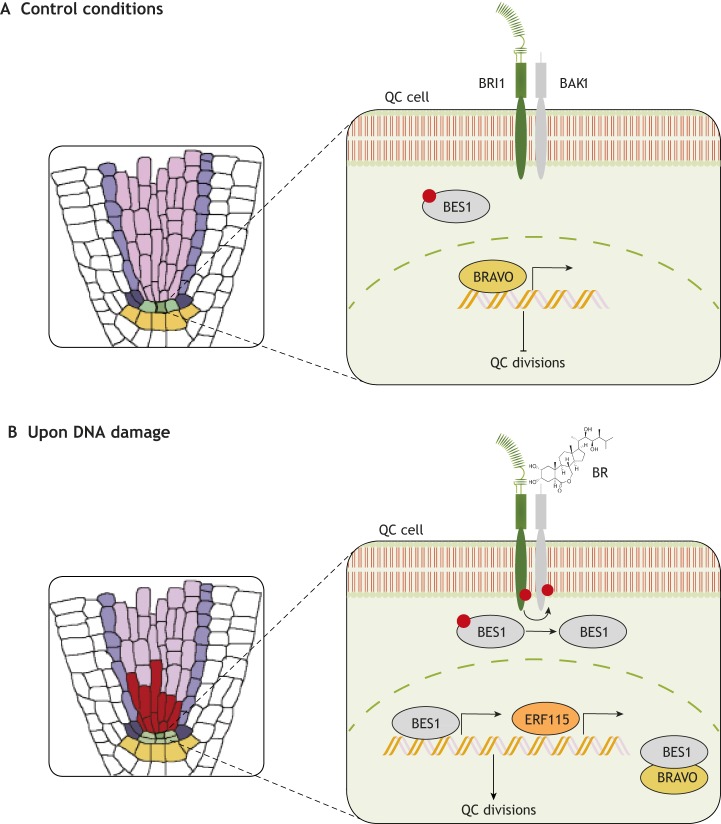
**Functional role of BRs in stem cell regeneration.** (A) In normal (‘control’) conditions, BR signaling in the QC is not active. This maintains BES1 in a phosphorylated and inactive state, permitting BRAVO to act and repress QC divisions. (B) In the presence of DNA damaging agents, vascular cells die and produce a local pool of BR that is sensed by BRI1/BRLs in a paracrine way in the QC. This leads to the dephosphorylation of BES1, the inactivation of BRAVO (both transcriptionally and via BRAVO heterodimerization with BES1) and the induction of ERF115 expression, which together promote the division of QC cells to replenish the dying cells.

BR signaling acts within the root stem cell niche by modulating BRAVO (BRASSINOSTEROIDS AT VASCULAR AND ORGANIZING CENTER) (Vilarrasa-Blasi et al., 2014). This transcription factor, also named MYB56, belongs to the R2R3-MYB family and is expressed specifically in vascular initials and QC cells (Vilarrasa-Blasi et al., 2014). Phenotypic analyses have shown that BRAVO represses QC cell divisions (Fig. 3A), as *bravo* mutants show a significant increase in QC division frequency. However, when BR signaling is activated, for example following DNA damage (Fig. 3B), the BR downstream effector BES1 becomes activated and downregulates the levels of *BRAVO* transcript. It also heterodimerizes with BRAVO protein itself, strongly inhibiting its action and promoting the division of QC cells (Vilarrasa-Blasi et al., 2014). This constitutes a regulatory circuit that controls QC division via interactions at both the transcriptional and protein levels. Another transcription factor that acts as a co-repressor of BRAVO is TPL (TOPLESS), which can bind to the *BRAVO* promoter as well as interact with BES1 via its ERF-associated amphiphilic repression (EAR) motif (Espinosa-Ruiz et al., 2017). Future studies aiming to dissect the cell-specific gene regulatory networks controlled by BRAVO in the stem cell niche will be instrumental for uncovering how and when QC cells divide.

BRs can also induce the expression of ERF115 (ETHYLENE RESPONSE FACTOR 115), a transcription factor that belongs to the ethylene response factor family and plays a key role in root growth and development. Specifically, ERF115 acts as a limiting factor for QC divisions as it regulates the expression of PSK5 (PHYTOSULFOKINES 5), a peptide hormone that enhances the frequency of QC divisions (Heyman et al., 2016, 2013). Collectively, BR signaling represses BRAVO activity and, at the same time, activates ERF115 to promote QC divisions when needed (Fig. 3B). However, it is still unknown where the signals that activate the BR pathway originate from, i.e. if they come from external tissues or if this process is carried out in a cell-autonomous way. Nonetheless, a recent study has shed some light on this matter, revealing that QC cell division is an autonomous process that needs BRI1 action within the stem cell niche (Lozano-Elena et al., 2018). This study also suggested that a paracrine signal leads to the activation of BES1 in QC cells in order to promote their division when needed. Thus, when the root suffers damage and stem cells undergo programmed cell death, the plant detects this scenario and starts promoting QC cell divisions to replenish damaged cells and to assure its survival. Although the mechanism underlying this response remains to be elucidated, it appears to involve a steroid paracrine signal from dead cells to the QC and that is perceived by BRI1 and transduced by BES1 (Lozano-Elena et al., 2018). We hypothesize that one such mobile signal could be the BR molecule itself, and that the increase in BR concentration in the stem cell niche could be due to a possible increase in BR biosynthetic genes, such as those encoding CPD and DWF4 (DWARF 4) (Lozano-Elena et al., 2018). However, further studies of BR synthesis and mobility are required to shed light on this matter (Vukasinovic and Russinova, 2018).

BRs also promote the differentiation of columella stem cells (CSCs), cells which are located distally to the QC. This occurs in a dose-dependent manner (González-García et al., 2011; Lee et al., 2015) via the transcription factor WOX5 (WUSCHEL-RELATED HOMEOBOX 5). WOX5 is a homolog of WUSCHEL, a transcription factor that maintains the identity of stem cells in the shoot (Mayer et al., 1998). In the root, WOX5 is required to maintain the identity of stem cells (Sarkar et al., 2007), and its transcript expression is restricted to the QC through external signals (Ding and Friml, 2010; Zhang et al., 2015) where it represses CYCD activity to establish quiescence (Forzani et al., 2014). *wox5* mutants show increased QC divisions and differentiated CSCs in the root apex (Sarkar et al., 2007). Importantly, the expression of WOX5 is regulated by BR; WOX5 expression decreases in *bri1-116* mutants (lacking the BRI1 receptor) and in plants treated with brassinazole (an inhibitor of BR biosynthesis). In contrast, WOX5 expression increases in plants treated with brassinolide (a bioactive form of BR) and in *bes1-D* or BRI1 overexpressor mutants (González-García et al., 2011).

In summary, BR levels are essential for regulating both cellular quiescence and the differentiation of stem cells in the root apex. Further studies, including cell-specific ‘omics’ approaches, will be key to decipher, for example, BRAVO partners and targets in the stem cell niche. It will also be interesting to decipher which receptors are involved in this context, and if the promotion of QC divisions is mediated primarily by BRI1 or if BRL1/3 play a major role as a consequence of their expression pattern throughout the root.

## Brassinosteroid signaling in adaptation to environmental stress

**Fig. 4.**
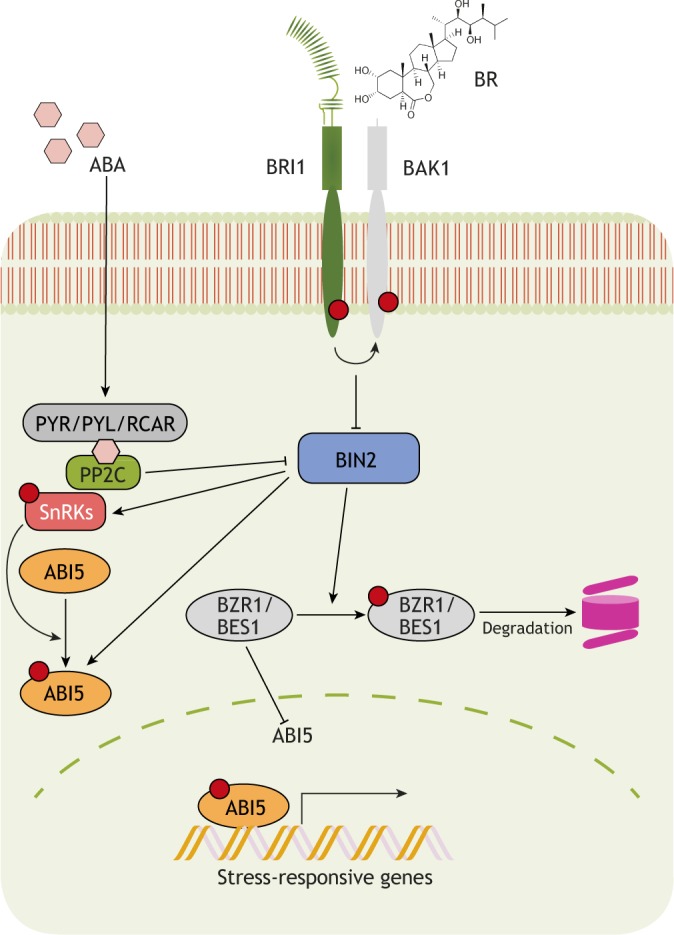
**BR-ABA crosstalk during the regulation of stress responses.** Schematic of the crosstalk between the BR and ABA pathways. ABA is perceived by PYR/PYL/RCAR receptors and promotes the phosphorylation and activation of SnRKs, thereby relieving them from PP2C-mediated repression. SnRKs, in turn, phosphorylate downstream transcription factors such as ABI5 that regulate the transcription of various stress-responsive genes. BIN2, which is a negative regulator of BR signaling, can also directly phosphorylate and activate SnRKs and ABI5, while PP2C is able to inactivate BIN2. ABI5 is also a direct target of BZR1, which represses its transcription to negatively regulate stress-responsive gene expression.

The ability of a plant to tolerate stress, such as changes in water availability, temperature or soil salinity, depends on its ability to switch between growth activation and repression in unfavorable conditions (Bechtold and Field, 2018; Feng et al., 2016). A key pathway that controls responses to environmental stresses is the abscisic acid (ABA) signaling pathway (Yoshida et al., 2014; Zhu et al., 2017). However, compelling evidence indicates that BRs also play a prominent role in controlling the balance between normal growth and resistance against environmental assaults, acting either via crosstalk with the ABA pathway or independently (Fig. 4). Several mechanisms have been proposed to explain how BR signaling mediates adaptation to stress. These include: (1) fine-tuning stress-responsive transcript machineries (Ye et al., 2017); (2) activating antioxidant machineries (Kim et al., 2012; Lima and Lobato, 2017; Tunc-Ozdemir and Jones, 2017; Xia et al., 2009; Zou et al., 2018); and (3) promoting the production of osmoprotectants (Fàbregas et al., 2018). As we discuss below, these various mechanisms contribute to BR-mediated adaptation to drought, cold, heat and salinity.

**Fig. 5.**
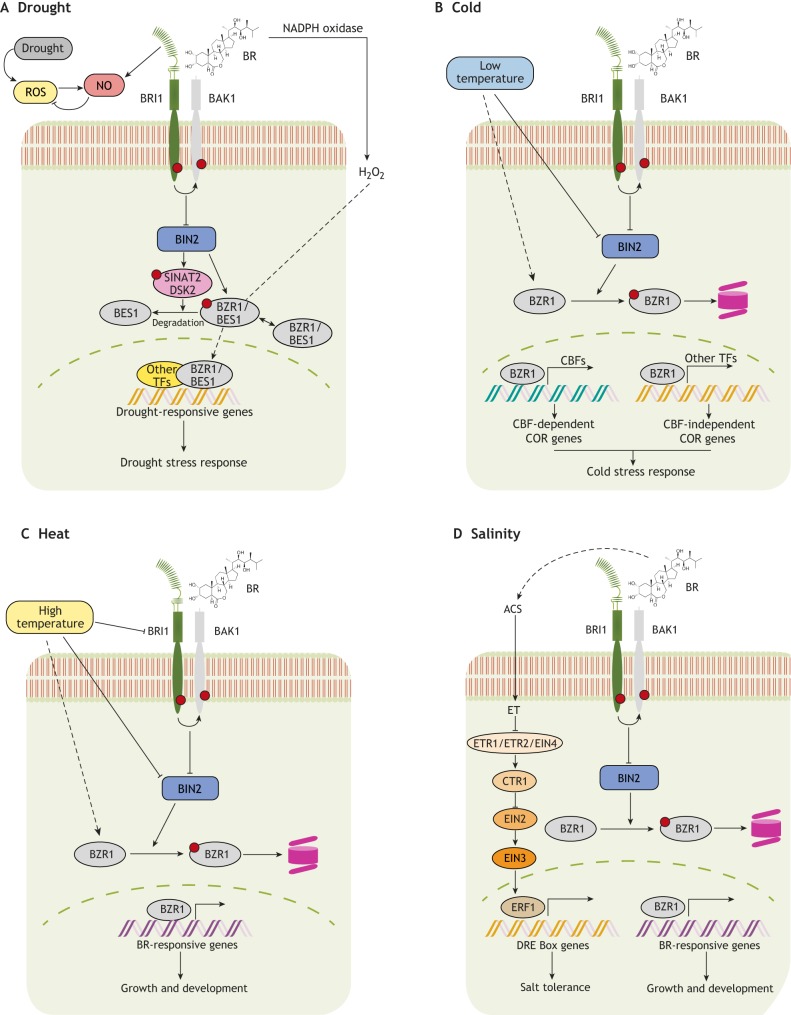
**BR signaling controls the switch between growth and abiotic stress responses.** (A-D) Schematics of cellular BR actions and crosstalk under conditions of drought (A), cold (B), heat (C) and high salinity (D). Notably, BRs act to control the balance between plant growth and stress responses. The BR and stress signaling pathways show multi-level crosstalk via their receptors, via the downstream kinase BIN2 and/or via transcription factors such as BZR1/BES1, depending on external as well as cellular environments. ET, ethylene.

BRs and ABA perform mostly antagonistic physiological functions, converging at the level of BIN2 and BZR1 (Cai et al., 2014; Hu and Yu, 2014). Whereas BIN2 acts a repressor of BR signaling (as discussed above), it enhances ABA-mediated stress responses by phosphorylating SnRK2 (SNF1-RELATED PROTEIN KINASE 2), leading to ABA-responsive gene expression (Cai et al., 2014). In addition, exogenous BR treatment inhibits the ABA-mediated induction of *RD26* (*RESPONSIVE TO DESICCATION 26*), a gene encoding a transcriptional activator of stress-inducible gene expression (Chung et al., 2014). This reciprocal antagonism between BR signaling and ABA-responsive transcription factors is key for coordinating plant growth and drought tolerance in *Arabidopsis* (Fig. 4). Indeed, it has been shown that RD26 is also a direct target of BES1 and is repressed by BR under drought conditions; reciprocally, RD26 modulates the transcription of BES1-regulated genes to inhibit BR function (Ye et al., 2017). The transcription factors WRKY46, 54 and 70 also interact with BES1 directly to promote BR-regulated plant growth while repressing drought-inducible global transcripts to inhibit drought tolerance (Chen and Yin, 2017). BIN2 phosphorylates and destabilizes WRKY54 to negatively regulate its effect on the BES1-mediated BR response (Chen et al., 2017). Recently, it was revealed that BR signaling via BIN2 interacts with autophagy pathways to coordinate plant growth and survival under drought stress and starvation (Nolan et al., 2017b) (Fig. 5A). In this context, BIN2 phosphorylates and activates the ubiquitin receptor protein DSK2, which further interacts with BES1 and targets it for degradation via autophagy (Nolan et al., 2017b). Together, these findings highlight the complexity of BR-mediated responses to drought. Future investigations are clearly needed to unravel the roles of individual BR signaling components and to understand how they switch the balance between normal versus drought-adapted growth and development.

BR signaling also modulates plant adaptation to different temperature stresses (Fig. 4B,C). The BR-regulated basic helix-loop-helix (bHLH) transcription factor CESTA activates the expression of C-REPEAT/DEHYDRATION-RESPONSIVE ELEMENT BINDING FACTOR (CBF) transcriptional regulators, which control the transcription of core cold responsive (COR) genes (Eremina et al., 2016). Another BR-regulated transcription factor, BR-ENHANCED EXPRESSION 1 (BEE1), promotes cold acclimation by indirectly influencing the transcription of MYB-bHLH-WD40 complex components (Petridis et al., 2016) (Fig. 5B). In line with these findings, BIN2 overexpression has been shown to cause hypersensitivity to freezing stress under both non-acclimated and acclimated conditions, whereas *bin2-3 bil1 bil2* triple mutants, as well as the gain-of-function *bzr1-1D* and *bes1-D* mutants, have enhanced tolerance for freezing stress (Li et al., 2017). BZR1 dephosphorylation is also induced upon cold treatment and can regulate COR genes, either directly or indirectly by binding to CBF1 and CBF2, and thereby affect the transcription of their downstream targets (Li et al., 2017) (Fig. 5B). BR signaling is also involved in regulating plant growth under high temperature stress (Fig. 5C). Upon elevated temperature, BZR1 accumulates in the nucleus and induces the expression of growth-promoting genes, either directly or via binding to the promoter of *PHYTOCHROME INTERACTING FACTOR 4* (*PIF4*) to regulate thermomorphogenesis (Ibañez et al., 2018; Oh et al., 2012). Elevated temperature has been shown to increase the accumulation of active PIF4, thereby shifting the balance of nuclear protein complexes towards BES1-PIF heterodimers instead of BES1 homodimers (Martinez et al., 2018). The subsequent reduced availability of active BES1 homodimers causes de-repression of BR biosynthesis and feedback inhibition of BR signaling output. In contrast, abundant levels of BES1-PIF4 complexes activate the genes involved in thermomorphogenesis (Martinez et al., 2018). Elevated ambient temperatures can also reduce BRI1 levels and affect primary root elongation growth (Martins et al., 2017). Recently, the kinase-defective BRI1 protein from *bri1-301* mutants was found to show less stability and biochemical activity under elevated temperature (29°C). A mutated version of this protein undergoes temperature-enhanced protein misfolding and degradation via an as-yet-unknown mechanism (Zhang et al., 2018). Together, these studies highlight a clear involvement of both BR receptors and downstream signaling components in regulating growth responses under fluctuating temperatures.

BR signaling is also able to mediate salt tolerance. It does so via the regulation of ethylene biosynthesis and signaling (Fig. 5D). Under salinity stress conditions, BR pre-treatment induces ethylene production, and hence signaling, by enhancing the activity of 1-aminocyclopropane-1-carboxylate synthase (ACS), an ethylene synthesis enzyme (Tao et al., 2015; Zhu et al., 2016). Conversely, blocking ethylene production and/or signaling components inhibits BR-induced antioxidant enzyme activities and salt tolerance (Tao et al., 2015; Zhu et al., 2016). The role for BR signaling in regulating salt stress tolerance may be mediated by BRI1; inhibiting the endoplasmic reticulum-associated protein degradation system is able to partially rescue the salt hypersensitivity of *bri1-9* mutants, providing evidence for the involvement of a membrane-bound BRI1 signaling complex in the salinity response (Cui et al., 2012). In contrast, *bin2-1* mutants are hypersensitive to salinity stress, and this correlates with inhibited induction of stress-responsive genes (Zeng et al., 2010). High salinity also causes growth quiescence in roots by suppressing nuclear accumulation of BZR1 and subsequent BR signaling functions (Geng et al., 2013). It is evident from the above-mentioned reports that exogenous application of BR helps plants to cope better under high salinity conditions by modulating both BR and ethylene signal outputs.

In addition to the crosstalk and mechanisms discussed above, the interplay between BR signaling and redox signaling appears to be crucial for plant development under stress (Fig. 5A). It is known that BR induces the antioxidant system during abiotic stress tolerance (Jiang et al., 2012; Zhou et al., 2014). BR has also been reported to utilize hydrogen peroxide (H_2_O_2_)- and nitric oxide (NO)-mediated mechanisms to provide stress tolerance (Cui et al., 2012; Xia et al., 2009). For example, during oxidative stress, BR increases ABA production through NO-mediated machinery (Zhang et al., 2011). BR-mediated transient H_2_O_2_ production via NADPH oxidase also triggers ABA biosynthesis, which, along with enhanced H_2_O_2_ production, acts as a positive-feedback mechanism for prolonged heat and oxidative stress tolerance (Zhou et al., 2014). The over-accumulation of superoxide anions (O_2_^−^) in the BR biosynthesis-defective mutant *det2-9* highlights yet another node of crosstalk between the BR and reactive oxygen species (ROS) pathways that is implicated in controlling root growth and development (Lv et al., 2018). Interestingly, this BR-mediated control of O_2_^−^ accumulation was found to occur through the peroxidase pathway rather than the NADPH oxidase pathway (Lv et al., 2018). H_2_O_2_-mediated oxidative modifications enhance the transcriptional activity of BZR1 and promote its interaction with ARF6 and PIF4. In contrast, the thioredoxin TRXh5 interacts with BZR1 and catalyzes its reduction (Tian et al., 2018) (Fig. 5A). Exogenous BR application also increases H_2_O_2_ production in the root stem cell niche, contributing to BR-induced QC division and cell elongation (Tian et al., 2018).

Nutrient availability in the soil microenvironment is another limiting factor for optimal root growth. BR signaling components were recently shown to regulate root growth behavior under low iron or phosphate levels (Singh et al., 2018). Specifically, it was found that BR signaling becomes activated upon iron deficiency and promotes root growth, and similarly that perturbed BR signaling affects iron distribution in *Arabidopsis* roots. In contrast, low phosphate levels cause enhanced iron accumulation, inhibiting BR signaling activation and subsequent root growth acceleration. The BRI1 negative regulator BKI1 was found to be the center point of this signal interplay, with BZR1/BES1, along with their direct target LPR1, which is a ferroxidase, acting at more downstream steps in this response (Singh et al., 2018). Moving forward, obtaining a more comprehensive understanding of the complex interplay between BR signaling, cellular redox status and the surrounding microenvironment will undoubtedly prove beneficial for understanding the mechanisms of plant survival and growth adaptation in suboptimal growth conditions.

Many of the BR-regulated stress adaptation responses discussed above have been described at the whole-plant survival level. However, recent technological advances are now allowing us to deconstruct the complexity of stress traits in a more spatiotemporal fashion. This approach has been instrumental in identifying the spatiotemporal roles of other phytohormones during stress responses in plants (Dinneny and Benfey, 2008; Geng et al., 2013; Iyer-Pascuzzi et al., 2011). Recently, a role for the vascular cell-specific activation of BR signaling in regulating drought adaptation in different developmental stages of root and shoot organs was uncovered using a multi-omics approach (Fàbregas et al., 2018). This study revealed that the quadruple BR receptor mutant (*bri1 brl1 brl3 bak1*) exhibits enhanced drought tolerance at the expense of overall growth. However, the overexpression of vascular-localized BRL3 receptors significantly improves drought tolerance without penalizing growth. In this case, BRL3 receptor accumulation in vascular tissues triggers the transcription of canonical water stress-response genes and osmoprotectant metabolism genes under both normal as well as water-deprived conditions. Metabolomic analyses confirmed that BRL3-overexpressing roots are enriched in osmoprotectant sugars and amino acids, and analysis of the transcriptome showed that it is enriched in genes involved in abiotic stress responses (Fàbregas et al., 2018). Altogether, these changes indicate that BRL3-overexpressing plants are better prepared for any upcoming stress, which in this case is drought (Fàbregas et al., 2018). This finding is corroborated by previous results reporting that BRs regulate metabolic flux, flavonol accumulation and anthocyanin synthesis during cold acclimation (Petridis et al., 2016), and other studies showing that BR application and BZR1 overexpression promote carotenoid, soluble sugar and ascorbic acid accumulation (Liu et al., 2014). Another example of spatiotemporal compartmentalization of BR signaling has recently been reported (Lozano-Elena et al., 2018). This study highlighted that paracrine BR signals from damaged cells can activate QC division and stem cell replenishment to compensate for root growth arrest upon genotoxic stress. Given the tissue-specific localization and regulation of different BR signaling components, combined with the complexity and diversity of stress-responsive mechanisms, it is likely that decisions of growth versus adaptation are made by signal activation/suppression on spatiotemporal scales. Understanding how these spatiotemporal variations in the activity of BRs control growth and plant adaptation to various environmental stresses is essential for understanding the mechanism by which plants balance growth with adaptation to ensure survival.

## Conclusions and perspectives

BRs are key for maintaining proper plant growth, both under normal conditions and in response to environmental stress, and ample evidence now supports the idea that modifying the BR response pathway can be a powerful strategy for designing better-adapted crops. However, our understanding of the main functions of BR signaling during stress is only generic, and the investigation of precise spatiotemporal- and context-specific regulatory mechanisms has only just begun (Kang et al., 2017; Lozano-Durán and Zipfel, 2015; Lozano-Elena et al., 2018; Vragović et al., 2015). Further studies are clearly required to obtain a more mechanistic understanding of the global and local actions of the BR pathway. With such knowledge, we could improve both the growth rates of plants and their adaptation to the environment by only changing the BR signal in specific tissues, making, for example, plants that are resistant to drought without altering their growth. Such an approach will be important to meet the food demands of an exponentially growing world population, especially when increasing plant yield in environmentally challenging conditions becomes essential (Food & Agriculture Organization, 2017).

**Fig. 6.**
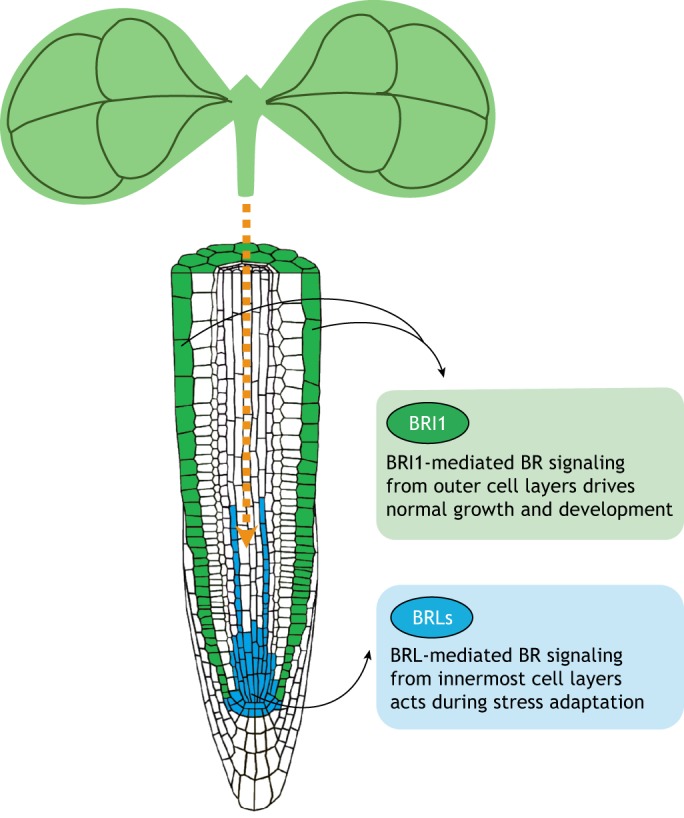
**Model depicting the tissue-specific actions of BR receptors during growth and stress responses.** Schematic of a scenario in which BRI1-based BR signaling from outer tissues (green) primarily regulates normal growth and development, whereas the signaling mediated by BRLs situated in the innermost cell layers (e.g. in the QC, the stem cell niche and vascular tissues; blue) controls stress adaptation responses. BRLs might also be involved in facilitating the mobilization of metabolic signals (orange arrow) from the shoot to root to provide stress tolerance.

Excitingly, studies have indicated that the local activities of the different BR receptors – BRI1 and the BRLs – and their effects on root development vary (Kang et al., 2017; Vragović et al., 2015); this could be one of the mechanisms through which BRs execute their pleiotropic effects on growth and stress adaptation. We propose a scenario (Fig. 6) in which, under normal conditions, BRI1-mediated signals drive the growth and development of roots and, subsequently, of the whole plant. This idea is supported by the finding that the lack of this receptor produces dwarf and sterile plants with shorter roots (Li and Chory, 1997). In contrast, the BRL1/BRL3 receptors seem to have little impact on these physiological processes, as mutants of both receptors (*brl1 brl3* mutants) do not show any visible phenotype (Caño-Delgado et al., 2004). However, these BR receptors, which exhibit tissue/cell-specific expression patterns, could be more involved during stress responses and adaptation. For example, vascular BRL3 expression is able to confer drought resistance, driving the accumulation of osmoprotectant metabolites in the root by promoting the activity of genes involved in their production. Moreover, it is known that *brl1 brl3* and *brl1 brl3 bak1* mutants have phenotypes associated with hydrotropism that are independent of the BRI1 pathway (Fàbregas et al., 2018), suggesting that the response to different stresses might be driven by BR receptors in specific cell types, such as stem cells and vascular tissues (Fig. 6). The identification of these BR receptor-driven differential signals will not only illustrate how different tissues coordinate their organ growth, but may prove to be useful for engineering new plants that have improved adaptation to the environment without modified growth.

Finally, it will also be important to capture the canonical as well as non-canonical signaling dynamics that function downstream of different BR receptor complexes. Examining these over different time and spatial scales may enable the identification of novel candidates that are relevant for adaptation upon stress-induced damage. A more precise and quantitative visualization of BRI1- and/or BRL-mediated cellular responses, such as ROS and NO production, stress-responsive transcription factor activation and downstream transcript regulation in different root tissues, will also help establish how BR executes stress protection and subsequent growth recovery. Overall, studies of the mechanisms underlying BR-regulated growth, in both optimal and stress conditions, will bring us closer to understanding the trade-off between growth and adaptation, and will help us strategize new approaches for creating smart root systems with efficient water and nutrient uptake abilities that can sustain crop biomass and yield.
